# Modulated scattering technique in the terahertz domain enabled by current actuated vanadium dioxide switches

**DOI:** 10.1038/srep41546

**Published:** 2017-02-01

**Authors:** W. A. Vitale, M. Tamagnone, N. Émond, B. Le Drogoff, S. Capdevila, A. Skrivervik, M. Chaker, J. R. Mosig, A. M. Ionescu

**Affiliations:** 1 EPFL, Nanoelectronic Devices Laboratory (NanoLab), 1015 Lausanne, Switzerland; 2 EPFL, Laboratory of Electromagnetics and Antennas (LEMA), 1015 Lausanne, Switzerland; 3 INRS-Énergie, Matériaux et Télécommunications, 1650 Boulevard Lionel Boulet, Varennes, Québec J3X 1S2, Canada

## Abstract

The modulated scattering technique is based on the use of reconfigurable electromagnetic scatterers, structures able to scatter and modulate an impinging electromagnetic field in function of a control signal. The modulated scattering technique is used in a wide range of frequencies up to millimeter waves for various applications, such as field mapping of circuits or antennas, radio-frequency identification devices and imaging applications. However, its implementation in the terahertz domain remains challenging. Here, we describe the design and experimental demonstration of the modulated scattering technique at terahertz frequencies. We characterize a modulated scatterer consisting in a bowtie antenna loaded with a vanadium dioxide switch, actuated using a continuous current. The modulated scatterer behavior is demonstrated using a time domain terahertz spectroscopy setup and shows significant signal strength well above 0.5 THz, which makes this device a promising candidate for the development of fast and energy-efficient THz communication devices and imaging systems. Moreover, our experiments allowed us to verify the operation of a single micro-meter sized VO_2_ switch at terahertz frequencies, thanks to the coupling provided by the antenna.

An electromagnetic scatterer is a structure able to scatter an impinging electromagnetic wave in various directions. Any object with electromagnetic properties different from the surrounding environment behaves as a scatterer. The modulated scattering technique (MST) is based on the modulation of the scattered field, which can be done by a mechanical change in the scatterer, or electronically using modulated scatterers (MS), which are linear passive electromagnetic devices able to control and change their electromagnetic scattering properties by embedding a tunable element^1^.

The operating frequency of MS devices has been in the range of hundreds of kHz up to millimeter waves, while THz frequency range has not been explored yet. The terahertz domain, conventionally defined as the portion of the electromagnetic spectrum included between 0.3 THz and 3 THz, is currently a very active research topic for various applications, such as telecommunications, imaging, spectroscopy, radioastronomy and homeland security. However, a widespread diffusion of THz systems has been hindered by the relative lack of available devices to generate, manipulate and detect THz waves. Here we perform a step forward in this direction by proposing the first MS operating in the THz range.

Among other applications, MS have been extensively used for field mapping applications^1^ and in radio frequency identification devices (RFID)^2^,^3^, where the technique is used to encode information in the scattered signal by the device when interrogated with a single frequency harmonic impinging wave. This is achieved by time-modulation of the tunable element, which modulates the scattered field propagating away from the device. Recently, MS were also considered as an important alternative for phase far-field imaging applications^4^,^5^. As compared with classical imaging techniques based on bolometers, which are limited to amplitude information, MST allows measuring both the amplitude and the phase of an electromagnetic wave at a single point on the sensor. This could be of great interest while considering an array of sensors, since having full knowledge of magnitude and phase for each pixel of the array would allow further processing not possible with only the knowledge of the received power (e.g. it could allow to re-focus the signal in a post-processing stage). Finally, single MS are very promising candidates for near-field imaging in the THz domain. Near-field imaging is already an extensively used technique at microwave frequencies^6^,^7^ and our work opens the way to the implementation of this paradigm at THz frequencies. Moreover, since MST operates with a differential signal, this technique allows greater accuracy than fixed scatterer near-field imaging. The achievement of MST in the THz domain could thus lead to interesting new strategies to perform amplitude and phase imaging at THz frequencies. In this work, we explore the feasibility of a THz modulated scatterer using a wideband antenna and a vanadium dioxide (VO_2_) switch as the tunable element (Fig. 1).

VO_2_ is a strongly correlated material that undergoes a first-order phase transition from a low-temperature insulating state to a high-temperature metallic state when heated above its transition temperature (~340 K)^8^. This phase transition, which is accompanied by a structural change from monoclinic to tetragonal and a large variation of VO_2_ electrical and optical properties^9^, can also be triggered using electrical^10^ or optical^11^ excitations, which makes this material an excellent candidate for electronic and optical switching applications^12^,^13^. The VO_2_ property exploited in this work is the steep reduction of its resistivity under electrical excitations. The proposed THz MS consists in a planar wideband bowtie metal antenna fabricated on a sapphire substrate, showing excellent transparency at THz frequencies, coated with a 500 nm VO_2_ layer. The small gap in the center of the antenna ensures that when a DC current is applied along the two arms of the antenna, the VO_2_ region in the antenna gap undergoes the phase transition and the scattering properties of the antenna are significantly altered due to the large decrease in the resistance of the switch. The exact nature of the physical mechanisms underlying the electrically-triggered metal-insulator transition in VO_2_ is still under debate. However, it is generally agreed that the transition cannot rely solely on the thermal heating effect, as demonstrated by several works reporting switching times of the order of few nanoseconds^14^,^15^,^16^.

It must be noticed that single VO_2_ switches were not previously exploited for THz reconfigurable electronics. While such switches were already characterized for radiofrequency^17^ and millimeter-wave^18^ applications, the use of VO_2_ in the THz range has been limited to the fabrication of metasurfaces in which the actuation of an unpatterned VO_2_ film allows tuning the polarization^19^,^20^, transmission^21^,^22^ or resonance frequency^23^,^24^ of the metasurface. The use of a single VO_2_ switch in our device is crucial, as it ensures operation even when the interrogation field and the receiver are placed at arbitrary angles with respect to the modulated scatterer, which is not the case for metasurfaces, optimized to preserve the propagation direction of the modulated wave. Thanks to this property, the MS device proposed in this work enables new functionalities at THz frequencies, complementary to what is achievable with metasurfaces. Furthermore, the use of a single VO_2_ switch reduces the power consumption and the response time of the device. Apart from modulation, it is worth mentioning that VO_2_ has a promising technological potential for THz detection and generation applications, allowing the development of VO_2_-based THz future front-end systems. For instance, THz generation from VO_2_ has been achieved via optical non-linearities^25^ or transient photocurrent contribution^26^.

## Results

### Design and numerical simulations

The design procedure consists in optimizing the MS antenna and the VO_2_ switch in order to maximize the power of the modulated signal *P*_mod_. This is achieved by maximizing the difference in the scattered field in the two states of the MS. The MST theory is well known and demonstrates that the power of the modulated signal can be calculated using the modified Friis link budget equation^1^:


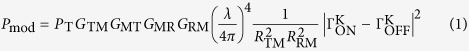


where *P*_T_ is the power emitted by the transmitter, *G*_TM_ and *G*_RM_ are the gains of the transmitting and receiving antennas towards the MS respectively, *G*_MT_ and *G*_MR_ are the gains of the MS antenna towards the transmitting and receiving antennas respectively, *R*_TM_ and *R*_RM_ are the distances from the MS to the transmitting and receiving antennas respectively, *λ* is the wavelength and Γ^K^ is known as Kurokawa’s reflection coefficient. Γ^K^ expresses the mismatch between the load and the antenna and it is defined as:


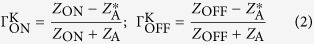


where *Z*_A_ is the input impedance of the MS antenna while *Z*_ON_ and *Z*_OFF_ are the impedances of the tunable load (here the VO_2_ switch with length *L* = 2 μm, width *W* = 4μm and VO_2_ thickness *t* = 500 nm) in the ON and OFF states respectively.

Equation 1 shows that *P*_mod_ depends on the modulation coefficient ΔΓ^K^, defined as the difference of the reflection coefficients 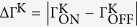, which can be maximized by optimizing the antenna input impedance and the VO_2_ switch impedance. Typical broadband bowtie antennas have input impedances with a constant real part (typically of the order of tens of ohms depending on both substrate and geometry) and a small imaginary part over the frequency band of interest. On the other hand, the VO_2_ switch impedance is dominated by carrier dynamics in VO_2_, which is approximately the same as in DC transport since the collision frequency is of the order of tens of terahertz^27^,^28^,^29^, as confirmed by direct measurements of the THz conductivity showing negligible imaginary part and a real part with low dependence on frequency^30^. Hence, the VO_2_ switch can be considered as resistive, i.e. its impedance is real, and its value can be computed from the bulk conductivity of VO_2_ as 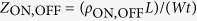. Therefore, ΔΓ^K^ is maximized if 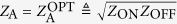
1. Typical VO_2_ switches have impedances of a few ohms in the ON state and of tens or even hundreds of kilohms in the OFF state, hence 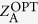 is expected to be of the order of kilohms. The antenna was thus optimized to maximize its input impedance while the switch was optimized to minimize 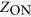. For this reason a relatively large thickness *t* = 500 nm of VO_2_ was used. Finally, using *ρ*_OFF_ = 4.7 · 10^−1^ Ω·m and *ρ*_ON_ = 4.3 · 10^−6^ Ω·m as obtained from four-point probe measurements, the load impedances are *Z*_OFF_ = 4.7 · 10^5^ Ω and *Z*_ON_ = 4.3 Ω. The full resistivity dependence on temperature of the VO_2_ film exploited in this work is reported in Supplementary Fig. S1.

**Figure 2:**
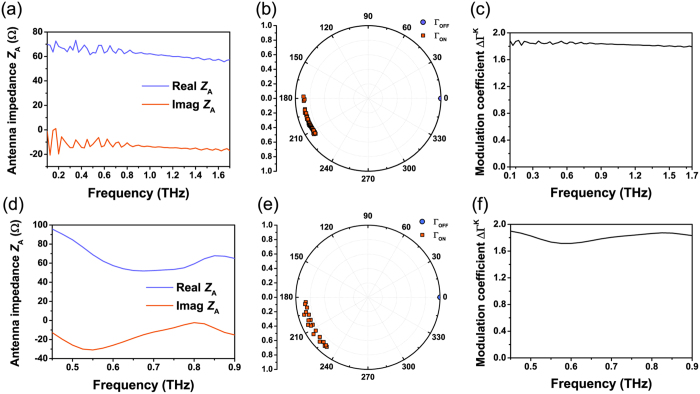
Simulations of THz modulated scatterers. (**a**) Input impedance *Z*_A_ for the antenna with optimized dimensions (*L*_A_ = 300 μm, *W*_A_ = 400 μm), simulated from 0.1 THz to 1.7 THz. (**b**) Reflection coefficient Γ^K^ for ON and OFF states for all the frequency points, obtained from the simulated *Z*_A_ values and the load impedance values for a VO_2_ switch with length *L* = 2 μm, width *W* = 4 μm and resistivities *ρ*_OFF_ = 4.7 · 10^−1^ Ω·m, *ρ*_ON_ = 4.3 · 10^−6^ Ω·m, extracted from four-point probe measurements of the VO_2_ film used in this work. (**c**) Resulting modulation coefficient. (**d**) Input impedance *Z*_A_ for the antenna with reduced dimensions (*L*_A_ = 60 μm, *W*_A_ = 80 μm), simulated from 0.45 THz to 0.9 THz. (**e**) Reflection coefficient Γ^K^ for ON and OFF states for the smaller MS keeping the same VO_2_ switch (*L* = 2 μm, *W* = 4 μm, *ρ*_OFF_ = 4.7 · 10^−1^ Ω·m, *ρ*_ON_ = 4.3 · 10^−6^ Ω·m). (**f**) Modulation coefficient for the smaller MS.

The antenna optimization was performed by 3D electromagnetic simulations using Ansys HFSS and resulted in an impedance *Z*_A_ independent of frequency from 0.1 THz to 1.7 THz with low imaginary part (Fig. 2(a)) for an antenna with dimensions *L*_A_ = 300 μm, *W*_A_ = 400 μm. Introducing the values for *Z*_ON_ and *Z*_OFF_ previously obtained and the antenna impedance *Z*_A_ in Eq. (2), we obtain a high contrast between 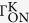 and 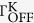 (Fig. 2(b)), resulting in a differential reflection coefficient ΔΓ^K^ ~ 1.8 in the whole frequency range (Fig. 2(c)), which constitutes an excellent performance considering that the maximum possible value is 2. Moreover, the design can be scaled to smaller dimensions while keeping the same *W*_A_/*L*_A_ ratio, enabling the use of MS as a small near-field probe of circuits or antennas^1^. Figure 2(d–f) present the results of the simulations for a smaller antenna in a range of THz frequencies such that 0.3 < *D*/λ < 0.6, where λ is the wavelength and *D* is the diameter of the smallest circumference that can circumscribe the antenna. This condition is satisfied from 0.45 THz to 0.9 THz for an antenna with *W*_A_ = 80 μm, *L*_A_ = 60 μm, resulting in *D* = 200 μm. As shown in Fig. 2(d), even in the case of an electrically small antenna, the bowtie design allows to achieve an impedance with a low imaginary part and a real part presenting low dependence on frequency, allowing to achieve a high difference in reflection coefficients (ΔΓ^K^ > 1.7, Fig. 2(f)) and, as a consequence, to maximize the power of the modulated signal. If further antenna miniaturization is required for any targeted application, the antenna can be modified using well known miniaturized antenna designs; however the improvement in miniaturization implies either a reduction in bandwidth or an increase in the losses of the antenna, in accordance with the Chu-Harrington limit^31^.

**Figure 3:**
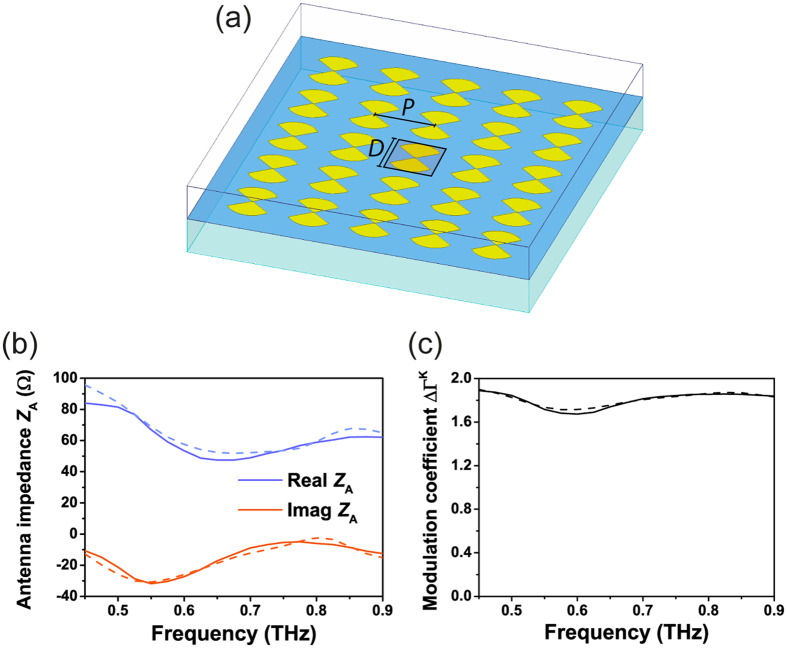
MS array simulations. (**a**) Full geometry domain for the electromagnetic simulation of an array of 5 × 5 MS (*L*_A_ = 60 μm, *W*_A_ = 80 μm, *D* = 200 μm, *P* = 250 μm), highlighting the MS for which the results are plotted in the following. (**b**) Antenna input impedance and (**c**) achieved modulation coefficient from 0.45 THz to 0.9 THz for the MS in the center of the array (solid lines) and an isolated MS with the same dimensions (dashed lines).

Further simulations were performed to address the feasibility of imaging applications based on the MST. Complex imaging involves heavy processing of the near-field information (magnitude and phase) in a given area of space, which can be provided with the use of MS arrays that would sample the field at the different positions of each MS probe. For this reason, we used the small MS sensor (*D* = 200 μm) to simulate an array of 5 × 5 MS, as shown in Fig. 3(a). The pitch *P* = 250 μm between the centers of the antennas is selected in order to have *P*/λ = 0.5 and *D*/λ = 0.4 for a frequency *f*_0_ = 0.6 THz. As shown in Fig. 3(b), the presence of additional sensors is not altering significantly their broadband performance as compared to a single antenna. As a consequence, ΔΓ^K^ is still higher than 1.65 in the whole frequency range of interest (Fig. 3(c)), demonstrating that the use of the proposed MST with a VO_2_ junction can be extended to an array without altering its design.

### Measurements

**Figure 4:**
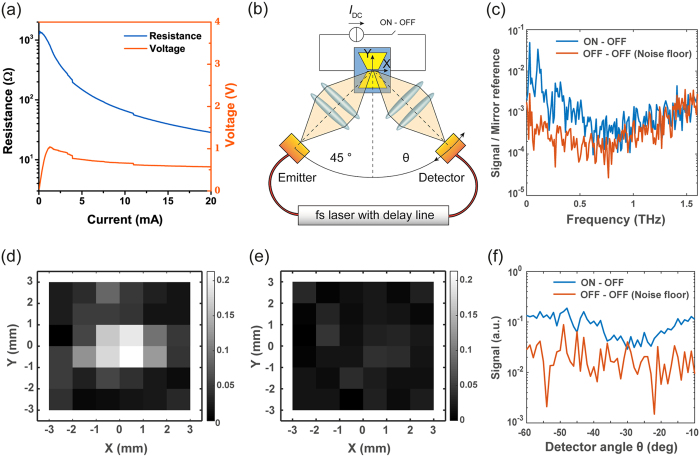
MS characterization. (**a**) DC characterization of the VO_2_ switch. (**b**) Schematic diagram of the THz measurement setup, including the rotary stage for the detector arm at angle θ, the imaging stage to move the MS in the XY plane and the DC biasing scheme with direct current *I*_DC_ = 20 mA. (**c**) THz differential signal normalized to the mirror reference with 10^3^ measurements cycles averaging and the receiving detector at −10°. (**d**) Differential signal and (**e**) noise floor imaging at 0.32 THz with 10^2^ cycles averaging while the setup is focused on the center (X = 0 mm, Y = 0 mm) and the MS is moved in a 6 × 6 mm^2^ area around the center. (**f**) Scattering pattern at 0.32 THz with 10^2^ cycles averaging.

The fabricated MS with optimized dimensions (*L* = 2 μm, *W* = 4 μm, *L*_A_ = 300 μm, *W*_A_ = 400 μm) was first measured in DC in order to characterize the electrical actuation of the VO_2_ switch integrated in the THz antenna. All the measurements were performed at room temperature. Figure 4(a) shows the resistance modulation of the VO_2_ switch due to the increase in direct current up to 20 mA. The measured resistance in the OFF state is 1.25 kΩ, which is considerably lower than that used in the design due to the current leakage flowing in the VO_2_ material outside the antenna gap: the parasitic resistance between the bias lines is in parallel to the modulated VO_2_ resistance inside the gap, decreasing the measured *Z*_OFF_ value. However, the performance of the THz MS is not affected, as it solely depends on the modulation of the impedance of the region inside the gap. The measured *Z*_OFF_ value thus represents a pessimistic estimate for the *Z*_OFF_ design parameter used in eq. (2) to calculate 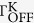. The resistance in the ON state depends on the bias current and it is 149 Ω at 5 mA and 28.5 Ω at 20 mA. In order to limit the power consumption and prevent reliability issues, the current was limited to 20 mA while operating the device, which was not high enough to reach the *Z*_ON_ value used in the design, but still low enough to ensure a good modulation coefficient (see Supplementary Fig. S2). The measured *Z*_ON_ value includes the finite contact resistance between VO_2_ and gold (Au) metal contacts^16^,^32^, whose effect is reduced at THz frequencies due to the parasitic contact capacitance in parallel.

Next, the operation of the device as a MS was proven using a fiber coupled time-domain THz spectroscopy system (Menlo TERA K15) in the configuration represented in Fig. 4(b). The transmission arm, which includes the THz emitter and a focusing lens, is placed at 45 degrees with respect to the sample normal, while the receiver arm, which includes another focusing lens and the detector, is mounted on a rotary stage at an angle θ with respect to the normal, allowing to perform scattering pattern measurements. The arm lenses lie at focal distance from the antenna to improve the signal-to-noise ratio (SNR) and the polarization of the THz beam is aligned to the polarization of the antenna (vertical polarization). The sample is glued and wire-bonded to a printed circuit board and mounted on XY imaging stages. All the measurements were performed at room temperature. The differential signal is obtained by subtracting the received field when the VO_2_ switch is in the OFF state from the one measured when the switch is ON (*I*_DC_ = 20 mA). The reference noise floor is obtained by measuring a second time the MS in the OFF state and subtracting it from the previous OFF state. All the measurements are normalized to the power of the THz pulse, obtained by measuring the received signal reflected by a mirror. The measurements were repeated for several cycles and averaged.

Figure 4(c) shows the received differential signal averaged for 1000 cycles as a function of frequency. The differential signal is well above the noise floor in the THz range and the maximum operation frequency is larger than 0.5 THz (see Supplementary Fig. S3 for the error bounds on the average signal). The decrease in performance with frequency is expected because of the imperfect focusing of the THz system, which limits the electromagnetic power coupled to the VO_2_ switch at high frequency. Some important considerations can be made on the unusual noise floor signal in Fig. 4(c). This signal is actually the difference of two quantities whose values are expected to be identical and, therefore, it represents both the random and the systematic errors. However, because the signal is normalized to the mirror reference, its shape is not monotonic and it instead represents the inverse of the SNR of our measurement setup, which is maximal at approximately 0.6 THz and decreases for higher and lower frequencies. Figure 4(d) shows an image of the differential signal averaged over 100 cycles obtained by moving the sample in the XY plane with the imaging stage (the noise floor is shown in Fig. 4(e) for comparison). The measurements were performed at 0.32 THz, where we observed the maximum SNR. This image demonstrates that the source of the differential signal is indeed localized on the MS and not due to other effects, since we observe a signal above the noise floor only when the MS is aligned to the focus. Finally, the measured radiation pattern of the MS at 0.32 THz, obtained for different angles of the receiving arm (the transmission arm being kept at 45° from the normal) is shown in Fig. 4(f), indicating that operation is possible over a wide range of angles.

## Discussion

We demonstrated the modulated scatterer technique in the THz domain and used VO_2_ as a tunable material to enable device operation on a wide range of frequencies well above 0.5 THz. We were also able to verify the operation of a single μm-sized VO_2_ switch in the THz range due to the excellent coupling with the antenna. These achievements were made possible by efficiently exploiting the large and steep decrease of the VO_2_ switch resistivity across the metal-insulator transition, which enables the scattering properties of the antenna to be altered. Furthermore, the MS structure scatters the incident THz wave in a wide range of directions, which allows different applications with respect to standard VO_2_ metasurfaces. This device is thus a very promising candidate for the development of fast and energy-efficient THz communication applications and phase-resolved THz imaging systems.

## Methods

### VO_2_ deposition

A VO_2_ thin film of 500 nm thickness was grown on *r*-cut oriented sapphire substrates (Al_2_O_3_ (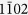)) by reactive pulsed laser deposition (RPLD) using a KrF laser (λ = 248 nm) at a repetition rate of 10 Hz. The vanadium metal target was ablated at a fluence of 2 J/cm^2^. Prior to deposition, the chamber was pumped down to 10^−6^ Torr. During deposition, the oxygen pressure was kept at 27 mTorr with a constant oxygen flow of 5 sccm. The target-to-substrate distance was set at 6.5 cm and the substrate temperature was maintained at 550 °C. Detailed growth conditions have been reported in a previous study^33^. The film thickness was determined by cross-section scanning electron microscopy (SEM) observations.

### MS fabrication

The THz bowtie antenna was fabricated on the top of the VO_2_ film by means of electron beam lithography (EBL). A 1 μm thick layer of poly(methyl-methacrylate) (PMMA) 950 K A7 from Microchem was first spin-coated on top of the VO_2_ film and baked at 180 °C for 5 minutes. The EBL was then performed at 100 keV and 60 nA using a VB6 UHR EWF (Raith Inc.), followed by the development of the exposed sample in a solution of methyl-isobutyl-ketone (MIBK) (1 vol.) and isopropyl alcohol (IPA) (3 vol.) for 75 s. Deposition of a 100 nm thick metal layer (90 nm Au)/(10 nm Cr) was performed after development by means of electron beam evaporation (K.J. Lesker AXXIS). The resist was then removed in dichloromethane and the sample was rinsed in acetone and IPA before being blown dry with pure nitrogen gas.

### Numerical simulations

The MS antennas were optimized by 3D full-wave electromagnetic simulations in the THz range, performed using the commercial simulation package Ansys HFSS. The boundaries of the simulation domain are at a distance from the antenna of at least λ_g_/4 in all directions, where λ_g_ is the wavelength in the medium, calculated at the minimum simulated frequency. The simulation was performed applying a lumped port in the gap between the two arms of the antenna. Radiation boundary conditions are applied to all the outer faces of the simulation domain. The relative permittivity of the sapphire substrate is ε_sap_ = 10 and the VO_2_ layer was assumed to have a relative permittivity in the insulating state ε_VO2_ = 30. Gold was simulated as a lossy conductor with conductivity σ_Au_ = 4.1 × 10^7^ S/m.

## Additional Information

**How to cite this article**: Vitale, W. A. *et al*. Modulated scattering technique in the terahertz domain enabled by current actuated vanadium dioxide switches. *Sci. Rep.* **7**, 41546; doi: 10.1038/srep41546 (2017).

**Publisher's note:** Springer Nature remains neutral with regard to jurisdictional claims in published maps and institutional affiliations.

## Supplementary Material

Supplementary Information

## Figures and Tables

**Figure 1:**
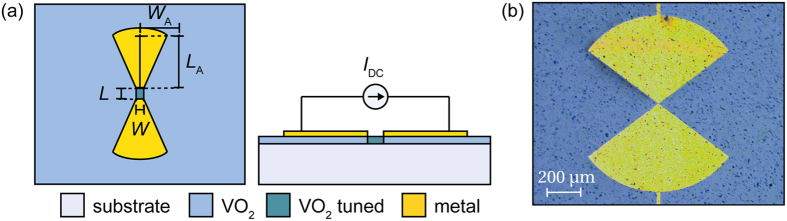
Structure of the proposed THz VO_2_ modulated scatterer. (**a**) Schematic diagram and biasing circuit. The bowtie antenna is connected to a DC current generator used to actuate the VO_2_ region in the antenna gap. (**b**) Top view of the fabricated device, with dimensions *L* = 2 μm, *W* = 4 μm, *L*_A_ = 300 μm, *W*_A_ = 400 μm, optimized to maximize the modulated signal.

## References

[b1] Bolomey J.-C. & Gardiol F. Engineering Applications of the Modulated Scatterer Technique. (Artech House, 2001).

[b2] Bolomey J. C., Capdevila S., Jofre L. & Romeu J. Electromagnetic Modeling of RFID-Modulated Scattering Mechanism. Application to Tag Performance Evaluation. Proc. IEEE 98, 1555–1569 (2010).

[b3] Capdevila S., Jofre L., Bolomey J.-C. & Romeu J. RFID Multiprobe Impedance-Based Sensors. IEEE Trans. Instrum. Meas. 59, 3093–3101 (2010).

[b4] Abou-Khousa M. & Zoughi R. Multiple Loaded Scatterer Method for E-Field Mapping Applications. IEEE Trans. Antennas Propag. 58, 900–907 (2010).

[b5] Ghasr M. T., Abou-Khousa M. a., Kharkovsky S., Zoughi R. & Pommerenke D. Portable Real-Time Microwave Camera at 24 GHz. IEEE Trans. Antennas Propag. 60, 1114–1125 (2012).

[b6] Dyson J. D. Measurement of Near Fields of Antennas and Scatterers. IEEE Trans. Antennas Propag. 21, 446–460 (1973).

[b7] Memarzadeh Tehran H., Laflamme-Mayer N., Laurin J. J. & Kashyap R. A near-field measurement setup using an array of optically modulated scatterers. in *Conference Proceedings of the International Symposium on Signals, Systems and Electronics* 481–484, doi: 10.1109/ISSSE.2007.4294518 (2007).

[b8] Morin F. J. Oxides which show a metal-to-insulator transition at the neel temperature. Phys. Rev. Lett. 3, 34–36 (1959).

[b9] Verleur H. W., Barker A. S. & Berglund C. N. Optical properties of VO_2_ between 0.25 and 5 eV. Rev. Mod. Phys. 40, 737 (1968).

[b10] Ruzmetov D., Gopalakrishnan G., Deng J., Narayanamurti V. & Ramanathan S. Electrical triggering of metal-insulator transition in nanoscale vanadium oxide junctions. J. Appl. Phys. 106 (2009).

[b11] Cavalleri A., Dekorsy T., Chong H. H. W., Kieffer J. C. & Schoenlein R. W. Evidence for a structurally-driven insulator-to-metal transition in VO_2_: A view from the ultrafast timescale. Phys. Rev. B - Condens. Matter Mater. Phys. 70, 1–4 (2004).

[b12] Zheludev N. I. & Kivshar Y. S. From metamaterials to metadevices. Nat. Mater. 11, 917–924 (2012). 23089997 10.1038/nmat3431

[b13] Yang Z., Ko C. & Ramanathan S. Oxide Electronics Utilizing Ultrafast Metal-Insulator Transitions. Annu. Rev. Mater. Res. 41, 337–367 (2011).

[b14] Leroy J. . High-speed metal-insulator transition in vanadium dioxide films induced by an electrical pulsed voltage over nano-gap electrodes. Appl. Phys. Lett. 100, 213507 (2012).

[b15] Zhou Y. . Voltage-Triggered Ultrafast Phase Transition in Vanadium Dioxide Switches. IEEE Electron Device Lett. 34, 220–222 (2013).

[b16] Joushaghani A. . Electronic and thermal effects in the insulator-metal phase transition in VO2 nano-gap junctions. Appl. Phys. Lett. 105, 231904 (2014).

[b17] Vitale W. A. . Steep slope VO_2_ switches for wide-band (DC-40 GHz) reconfigurable electronics. in *72nd Device Research Conference* 29–30, doi:10.1109/DRC.2014.6872284 (2014).

[b18] Hillman C., Stupar P. A. & Griffith Z. VO_2_ Switches for Millimeter and Submillimeter-Wave Applications. In 2015 IEEE Compound Semiconductor Integrated Circuit Symposium (CSICS) 1–4, doi: 10.1109/CSICS.2015.7314528 (IEEE, 2015).

[b19] Vegesna S. . Terahertz frequency selective surface with reconfigurable polarization characteristics using vanadium dioxide. J. Electromagn. Waves Appl. 28, 83–90 (2014).

[b20] Shin J.-H., Moon K., Lee E. S., Lee I.-M. & Hyun Park K. Metal-VO_2_ hybrid grating structure for a terahertz active switchable linear polarizer. Nanotechnology 26, 315203 (2015). 26183858 10.1088/0957-4484/26/31/315203

[b21] Jeong Y.-G. . Electrical control of terahertz nano antennas on VO_2_ thin film. Opt. Express 19, 21211–5 (2011). 22108973 10.1364/OE.19.021211

[b22] Jeong Y.-G. . A Vanadium Dioxide Metamaterial Disengaged from Insulator-to-Metal Transition. Nano Lett. 15, 6318–6323 (2015). 26352780 10.1021/acs.nanolett.5b02361

[b23] Driscoll T. . Dynamic tuning of an infrared hybrid-metamaterial resonance using vanadium dioxide. Appl. Phys. Lett. 93, 24101 (2008).

[b24] Zhu Y. . Tunable dual-band terahertz metamaterial bandpass filters. Opt. Lett. 38, 2382–4 (2013). 23939055 10.1364/OL.38.002382

[b25] Esaulkov M. . Emission of terahertz pulses from vanadium dioxide films undergoing metal–insulator phase transition. Optica 2, 790 (2015).

[b26] Charipar N. A., Kim H., Mathews S. A. & Piqué A. Broadband terahertz generation using the semiconductor-metal transition in VO_2_. AIP Adv. 6, 15113 (2016).

[b27] Wang H., Yang Y. & Wang L. Wavelength-tunable infrared metamaterial by tailoring magnetic resonance condition with VO_2_ phase transition. J. Appl. Phys. 116, 123503 (2014).

[b28] Hilton D. J. . Enhanced Photosusceptibility near T_c_ for the Light-Induced Insulator-to-Metal Phase Transition in Vanadium Dioxide. Phys. Rev. Lett. 99, 226401 (2007). 18233305 10.1103/PhysRevLett.99.226401

[b29] Peterseim T., Dressel M., Dietrich M. & Polity A. Optical properties of VO_2_ films at the phase transition: Influence of substrate and electronic correlations. J. Appl. Phys. 120, 75102 (2016).

[b30] Cocker T. L. . Terahertz conductivity of the metal-insulator transition in a nanogranular VO_2_ film. Appl. Phys. Lett. 97, 19–22 (2010).

[b31] Chu L. J. Physical Limitations of Omni-Directional Antennas. J. Appl. Phys. 19, 1163 (1948).

[b32] Joushaghani A. . Voltage-controlled switching and thermal effects in VO2 nano-gap junctions. Appl. Phys. Lett. 104, 221904 (2014).

[b33] Émond N., Hendaoui A. & Chaker M. Low resistivity W_x_V_1−x_O_2_-based multilayer structure with high temperature coefficient of resistance for microbolometer applications. Appl. Phys. Lett. 107, 143507 (2015).

