# Supplementary material for: Modulated scattering technique in the terahertz domain enabled by current actuated vanadium dioxide switches
Source: Sci Rep. 2017 Feb 1;7:41546. doi: 10.1038/srep41546 (PMC5286402; doi:10.1038/srep41546)
Supplement: Supplementary Information [file srep41546-s1.pdf]

Modulated scattering technique in the terahertz domain enabled by current actuated vanadium dioxide switches

W. A. Vitale,[1*] M. Tamagnone,[2*] N. Émond,[3] B. Le Drogoff,[3] S. Capdevila,[2] A. Skrivervik,[2] M. Chaker,[3] J. R. Mosig,[2] and A. M. Ionescu[1]

[1] *EPFL, Nanoelectronic Devices Laboratory (NanoLab), 1015 Lausanne, Switzerland*

[2] *EPFL, Laboratory of Electromagnetics and Antennas (LEMA), 1015 Lausanne, Switzerland*

[3] *INRS-Énergie, Matériaux et Télécommunications, 1650 Boulevard Lionel Boulet, Varennes, Québec, Canada J3X 1S2*

[*] *These authors contributed equally to this work*

Supplementary Information

Fig. S1 shows the electrical resistivity dependence on temperature of the VO_2 film exploited in this work. As expected, the resistivity value is almost temperature-independent in the metallic phase ($\rho_{ON} = 4.3 \cdot 10^{-6}$ Ω·m), whereas it decreases exponentially with temperature in the insulating phase. The resistivity value in the OFF state used in the design section was extracted at room temperature, $T = 25$ °C, resulting in $\rho_{OFF} = 4.7 \cdot 10^{-1}$ Ω·m.

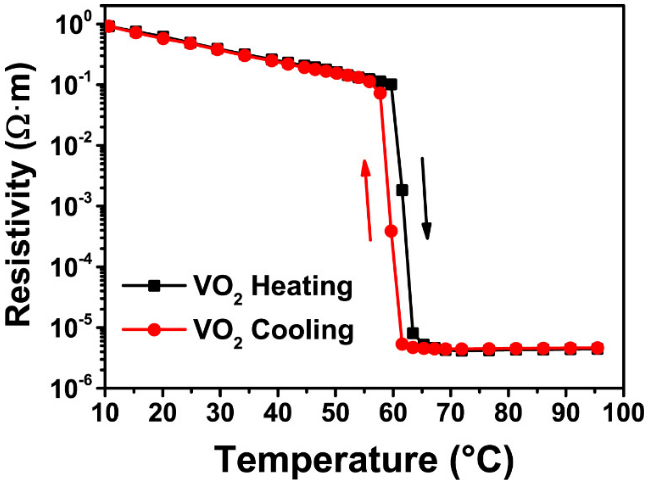

Figure S1. VO_2 electrical resistivity dependence on temperature. Dependence on temperature of the VO_2 resistivity while heating the film from 10 °C to 95 °C and cooling back to 10 °C.

Fig. S2 shows the effect of using the experimental, pessimistic values of Z_{OFF} and Z_{ON} on the estimated modulation coefficient $\Delta\Gamma^K$. In this worst-case condition we still achieve a modulation coefficient higher than 1.2 in the whole frequency range.

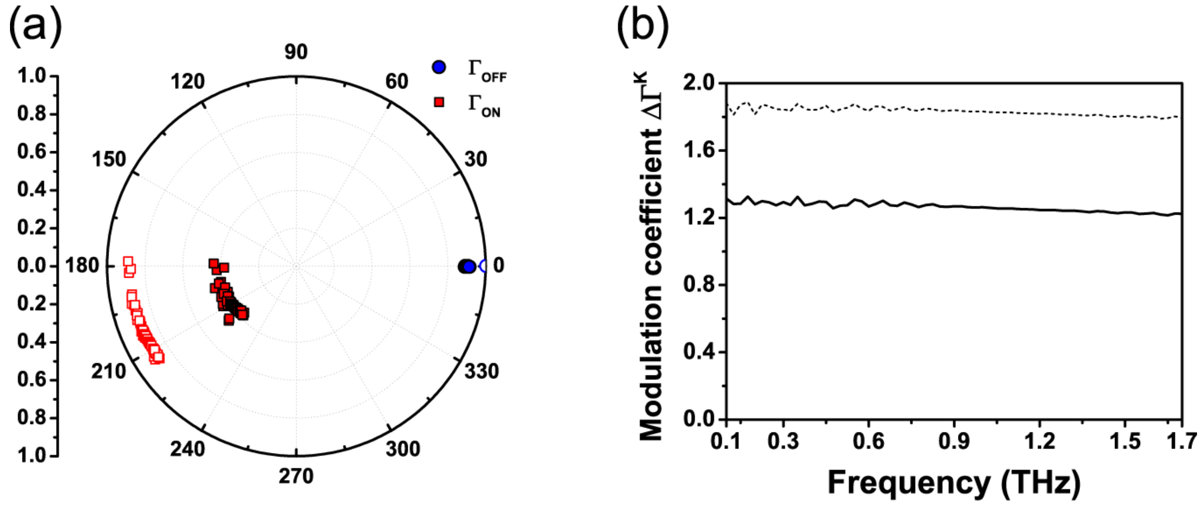

Figure S2. Simulations of THz modulated scatterers based on experimental values of Z_{OFF} and Z_{ON}. (**a**) Experimental (filled symbols) and design (empty symbols) values of the reflection coefficient Γ^K for ON and OFF states for all the frequency points, obtained from the simulated Z_A values and load impedance values for a VO_2 switch with length L = 2 µm and width W = 4 µm. (**b**) Resulting experimental (solid lines) and design (dotted lines) values of the modulation coefficient.

Fig. S3 and S4 show the error bounds for the THz differential signal, obtained adding and subtracting the standard error to the averaged signal. We can observe that the standard error of the signal is very limited up to 0.7 THz, with reliable MST operation possible for operating frequencies higher than 0.5 THz.

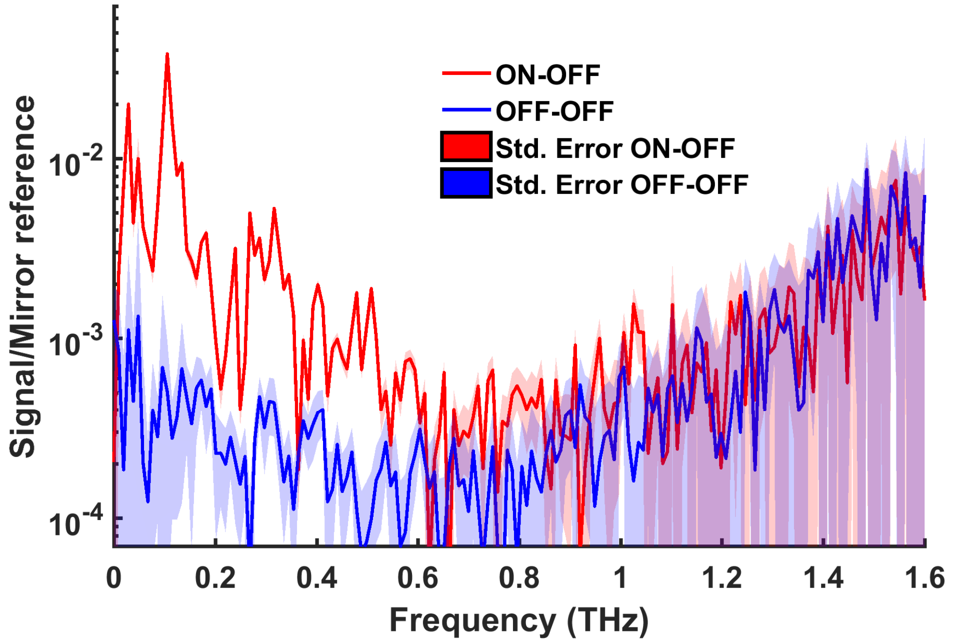

Figure S3. THz differential signal normalized to the mirror reference including error bounds. THz differential signal normalized to the mirror reference with 10^3 measurements cycles averaging and the receiving detector at -10°. Error bounds obtained adding and subtracting the standard error (also known as typical error) to the signal. The standard error is defined as the standard deviation of the measures divided by the square root of the number of samples, and it is computed for each frequency point.

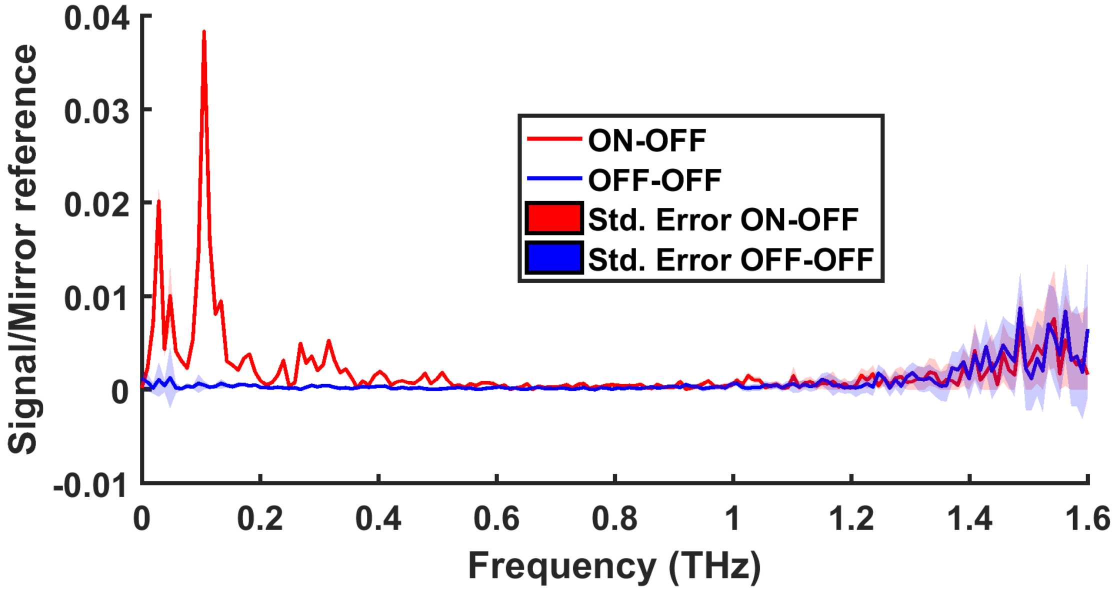

Figure S4. THz differential signal normalized to the mirror reference including error bounds, linear scale.
